# Embedded Weapons-Grade Tungsten Alloy Shrapnel Rapidly Induces Metastatic High-Grade Rhabdomyosarcomas in F344 Rats

**DOI:** 10.1289/ehp.7791

**Published:** 2005-02-15

**Authors:** John F. Kalinich, Christy A. Emond, Thomas K. Dalton, Steven R. Mog, Gary D. Coleman, Jessica E. Kordell, Alexandra C. Miller, David E. McClain

**Affiliations:** ^1^Heavy Metals Research Team and; ^2^Veterinary Sciences Department, Armed Forces Radiobiology Research Institute, Bethesda, Maryland, USA; ^3^Division of Veterinary Pathology, Walter Reed Army Institute of Research, Silver Spring, Maryland, USA

**Keywords:** cobalt, embedded fragment, nickel, rat, rhabdomyosarcoma, tungsten, tungsten alloy

## Abstract

Continuing concern regarding the potential health and environmental effects of depleted uranium and lead has resulted in many countries adding tungsten alloy (WA)-based munitions to their battlefield arsenals as replacements for these metals. Because the alloys used in many munitions are relatively recent additions to the list of militarily relevant metals, very little is known about the health effects of these metals after internalization as embedded shrapnel. Previous work in this laboratory developed a rodent model system that mimicked shrapnel loads seen in wounded personnel from the 1991 Persian Gulf War. In the present study, we used that system and male F344 rats, implanted intramuscularly with pellets (1 mm × 2 mm cylinders) of weapons-grade WA, to simulate shrapnel wounds. Rats were implanted with 4 (low dose) or 20 pellets (high dose) of WA. Tantalum (20 pellets) and nickel (20 pellets) served as negative and positive controls, respectively. The high-dose WA-implanted rats (*n* = 46) developed extremely aggressive tumors surrounding the pellets within 4–5 months after implantation. The low-dose WA-implanted rats (*n* = 46) and nickel-implanted rats (*n* = 36) also developed tumors surrounding the pellets but at a slower rate. Rats implanted with tantalum (*n* = 46), an inert control metal, did not develop tumors. Tumor yield was 100% in both the low- and high-dose WA groups. The tumors, characterized as high-grade pleomorphic rhabdomyosarcomas by histopathology and immunohistochemical examination, rapidly metastasized to the lung and necessitated euthanasia of the animal. Significant hematologic changes, indicative of polycythemia, were also observed in the high-dose WA-implanted rats. These changes were apparent as early as 1 month postimplantation in the high-dose WA rats, well before any overt signs of tumor development. These results point out the need for further studies investigating the health effects of tungsten and tungsten-based alloys.

Tungsten has been used for many years in a variety of applications. Combining the hard, brittle tungsten metal with various other metals, including nickel and cobalt, produces tungsten alloys (WAs) with specific characteristics, some of which are of interest to the military. Recently, WAs have replaced lead in some small-caliber ammunition (the “green bullet”) [Oak Ridge National Laboratory (ORNL) 1998] and depleted uranium (DU) in kinetic-energy penetrators (ORNL 1996). Based on a small number of studies, prevailing theory is that elemental tungsten or insoluble tungsten compounds have only limited toxicity (Leggett 1997). For example, tungsten coils implanted into the subclavian artery of rabbits rapidly degrade, leading to elevated serum tungsten levels as early as 15 min after implantation. However, after 4 months, no signs of local or systemic toxicity were observed (Peuster et al. 2003). Studies on health effects of Ni and Co are more numerous. Intramuscular injections (28 mg) of soluble metallic Ni or Co result in formation of rhabdomyosarcomas at the injection site. With Ni, 100% of injected rats develop a tumor within 41 weeks (Heath and Daniel 1964), whereas administration of Co results in tumor formation in 40% of the rats with a latency period of 71 weeks (Heath 1954, 1956). However, intramuscular implantation of rods or pellets composed of various Ni or Co alloys used in orthopedic prosthetics results in no excessive tumor formation (Gaechter et al. 1977; Sunderman 1989). A variety of other Ni compounds, including nickel subsulfide, nickel oxide, and nickel monosulfide, have been tested for carcinogenic potential via intramuscular administration (Gilman 1962; Sunderman and Maenza 1976; Sunderman et al. 1977). Tumors (rhabdomyosarcoma and fibrosarcoma) were found in many cases at the injection site, with tumor yield dependent on solubility and concentration of the administered compound. It has been postulated that the yield of localized tumors is inversely related to the rate of solubilization of the Ni-containing compound (Kasprzak et al. 1983). This hypothesis does not appear to hold for Co compounds (Lison et al. 2001).

Metal alloys present additional problems when investigating health effects. The various metals comprising the alloy, as well as the method of production, can all factor into the overall health effect observed upon exposure. Investigations on hard-metal disease have shown that either tungsten carbide or Co alone has limited toxicity on lung tissue (Lasfargues et al. 1992). However, when combined, the tungsten carbide/cobalt mixture acts synergistically to increase the observed toxicity. It is not known whether this is due to the combined toxicity of the tungsten carbide/cobalt mixture or to an increase in the bioavailability of the known toxicant, Co (Lison and Lauwerys 1997). *In vitro* studies investigating malignant transformation of immortalized human cells by mixtures of tungsten, Ni, and Co suggest a synergistic effect that greatly exceeds the effects of the metals individually (Miller et al. 2001, 2002).

Advancements in metallurgy have led the military of many nations to replace DU in some armor-penetrating munitions and lead in small-caliber ammunition with various alloys of tungsten. One motivation for such a replacement is widespread public concern about the health and environmental impact of continued use of these metals. However, to our knowledge, none of these militarily relevant WAs has been tested for potential health effects, especially as embedded shrapnel. There is a growing list of health concerns related to tungsten exposure. Although a definitive link has not been established, several cancer clusters in the United States are associated with elevated levels of tungsten in the environment. Those findings, along with the results presented in this article, raise questions about the possible consequences of tungsten exposure. More important, these results raise extremely serious concerns over the potential health effects of WA-based munitions currently being used as nontoxic alternatives to lead and DU.

## Materials and Methods

### Rodents.

Male F344 rats (6 weeks of age; Harlan, Frederick, MD) were maintained in a facility accredited by the Association of Assessment and Accreditation of Laboratory Animal Care in accordance with the *Guide for the Care and Use of Laboratory Animals* (Institute of Laboratory Animal Resources 1996). All procedures, including euthanasia criteria (Tomasovic et al. 1988), were approved by the Armed Forces Radiobiology Research Institute’s (AFRRI) Animal Care and Use Committee. Upon arrival, animals were screened for common rodent pathogens. Rats were pair-housed in plastic microisolator cages with hardwood chips for bedding and fed a certified NTP-2000 (Quality Lab Products, Elkridge, MD) diet (Rao 1996) with acidified water provided *ad libitum*. Animals were on a 12-hr light/dark cycle with no twilight and were weighed weekly.

### Pellets.

All metal pellets were cylinders 1 mm in diameter and 2 mm in length. Nickel (99.995% metallic Ni) and tantalum (99.95% Ta) pellets were purchased from Alfa Aesar (Ward Hill, MA). WA pellets were fabricated by Aerojet Ordnance Tennessee (Jonesborough, TN) using standard kinetic energy penetrator production processes. An average WA pellet weighed 27.5 mg and consisted of 91.1% tungsten, 6.0% Ni, and 2.9% Co. Ni and Ta pellets weighed 14 mg and 27 mg, respectively. Before implantation surgery, all pellets were cleaned and chemically sterilized (Pellmar et al. 1999).

### Pellet-implantation surgery.

A rodent model system (AFRRI 1996), originally developed to mimic DU shrapnel loads seen in wounded personnel from the 1991 Persian Gulf War, was used to investigate the health effects of retained WA shrapnel. All rats were implanted with a total of 20 pellets split evenly between each hind leg. Experimental groups included Ta (negative control, 20 Ta pellets), low-dose WA (4 WA pellets and 16 Ta pellets), high-dose WA (20 WA pellets), and Ni (positive control, 20 Ni pellets). Tantalum was used as a negative implantation control because it is considered inert and has been used in human prostheses (Hockley et al. 1990; Johansson et al. 1990). Nickel, a known carcinogen, was used as a positive control (Costa and Klein 1999; Kasprzak et al. 2003). Rats were implanted at 9 weeks of age. For the pellet implantation procedure, anesthesia was induced by continuous administration of isoflurane using an open circuit system with a scavenger/recapture system. All surgery was done using aseptic techniques. After the surgical sites were clipped and cleansed with Betadine, an incision was made through the skin to expose the gastrocnemius muscle. Pellets were implanted in the muscle, spaced approximately 1.5 mm apart on the lateral side of each leg. The incision was closed with sutures and tissue adhesive. Rats were closely monitored after surgery until they were ambulatory. An analgesic (buprenorphine hydrochloride; Reckitt and Colman, Hull, UK) was administered preoperatively and then as needed postoperatively. The surgical sites were examined daily for signs of inflammation, infection, and local metal toxicity.

### Experimental groups.

Our pellet implantation groups included Ta (negative control), WA (both a low- and high-dose group), and Ni (positive control). The original euthanasia time points were to be 1, 3, 6, 12, 18, and 24 months; however, because of the rapid tumor development, no WA- or Ni-implanted rat survived much past 6 months post-implantation. Final survival data therefore included rats originally assigned to the 12-, 18-, and 24-month experimental groups, whose animals died earlier than those designated time points. This resulted in group sizes of *n* = 46 for the Ta and both WA groups, and *n* = 36 for the Ni group. Hematologic assessments were conducted on the separate 1-, 3-, and 6-month WA implantation groups.

### Pathology.

At various times postimplantation or when moribund, rats were euthanized by isoflurane overdose. A complete gross pathology examination was conducted, noting any abnormalities, and tissues were collected for analysis. Weights of representative tissues, including spleen, thymus, testes, kidney, and liver, were determined and normalized to body weight. Tissues for histopathology were fixed in buffered formalin, processed and embedded in paraffin, cut at 5–6 μm, mounted, and stained with hematoxylin and eosin (H&E). Immunohistochemical analysis was conducted on 5-μm-thick sections of formalin-fixed, paraffinized tissue. After deparaffination and rehydration, nonspecific binding was blocked with Power Block (Biogenex, San Ramon, CA). The tissue was then reacted with prediluted rabbit anti-desmin polyclonal antibody (Biogenex) and treated with biotinylated secondary anti-rabbit antibody (Biogenex). After blocking with hydrogen peroxide, the tissue sections were labeled with peroxidase-conjugated streptavidin (Biogenex) and aminoethyl carbazole (AEC; Biogenex) was used as a chromogen. Slides were then counter-stained with hematoxylin and mounted.

### Hematology.

At euthanasia, we obtained blood for hematologic assessments from the abdominal aorta of isoflurane-anesthetized rats using a heparinized needle and sample tubes containing EDTA (Becton-Dickinson, Franklin Lakes, NJ). We determined white and red blood cell counts; hemoglobin; hematocrit; mean corpuscular volume, hemoglobin, and hemoglobin concentration; red cell distribution width; platelet counts and volume; and neutrophil, lymphocyte, monocyte, eosinophil, and basophil counts with a Bayer Advia 120 Hematology Analyzer (Bayer Diagnostics, Terrytown, NY).

## Results

All rats tolerated the pellet implantation procedure with no apparent adverse effects. The incision sites were examined daily; no rat showed any signs of infection from the surgery, or any discomfort postoperatively. Body weights were recorded weekly. Once they had recovered from the surgical procedure, all rats gained weight at equivalent rates. However, in the first week after the pellet implantation surgery, the rate of weight gain by the Ta and low-dose WA rats was slower than normal, and high-dose WA and Ni rats lost weight. This was followed by large weight gains in postimplantation week 2 in all experimental groups. There were no statistical differences in rate of body weight gain between any of the groups throughout the remaining experimental period. As previously reported, the implantation and retention of cylindrical metal pellets (1 mm ×2 mm) had no effect on locomotive abilities in rats (AFRRI 1996; Pellmar et al. 1999), nor did we observe any such difficulties in this study.

**Figure 1:**
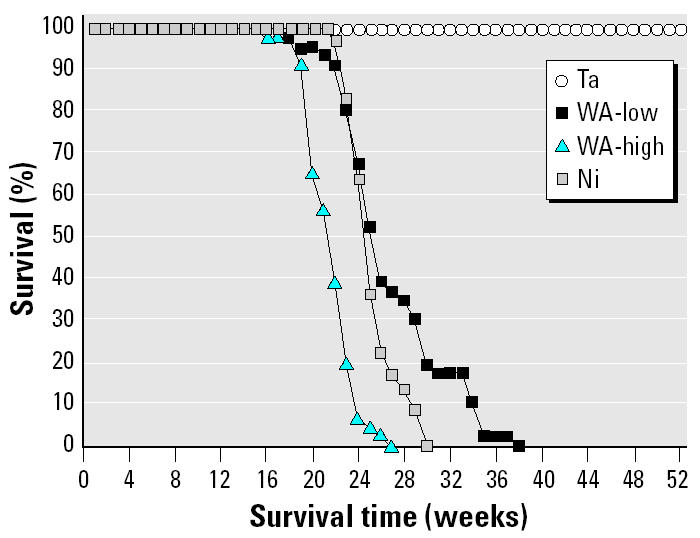
Survival times of pellet-implanted rats.

At approximately 16–20 weeks post-implantation, we began to observe tumors at the pellet implantation sites in the WA and Ni rats. In some high-dose WA animals, palpable tumors were apparent as early as 14 weeks postimplantation. Tumors developed rapidly in WA-implanted animals. The tumors were aggressive and fast growing, necessitating euthanasia of the animals several weeks later. On the basis of previously published literature (Heath and Daniel 1964), we expected the Ni-implanted positive control rats to develop tumors at the implantation site, but the speed at which the tumors developed was surprising: approximately 5 months after implantation. Figure 1 shows the percentage of surviving animals as a function of time after pellet implantation. Rats implanted with Ta pellets (*n* = 46) survived well beyond 12 months with no apparent health problems. All rats in the high- and low-WA and the Ni groups developed tumors and were euthanized upon becoming moribund. Rats in the high-dose WA group (*n* = 46) survived the least amount of time (mean survival time ± SD = 21.8 ± 2.1 weeks). Nickel-implanted animals (*n* = 36) and the low-dose WA group (*n* = 46) survived slightly longer, with mean (± SD) survival times of 25.4 ± 2.1 and 27.0 ± 4.6 weeks, respectively. The mean survival time of the high-dose WA animals was significantly shorter than that of the low-dose WA- or Ni-implanted animals [analysis of variance (ANOVA) followed by Dunnett’s test, *p* < 0.05]. The mean survival times of the low-dose WA- and the Ni-implanted animals were not statistically different from each other. The results reported here are part of a larger study that also investigated the health effect of embedded DU fragments. We did not observe tumor formation in the DU-implanted rats (Kalinich JF, Miller AC, McClain DE, unpublished data).

**Figure 2:**
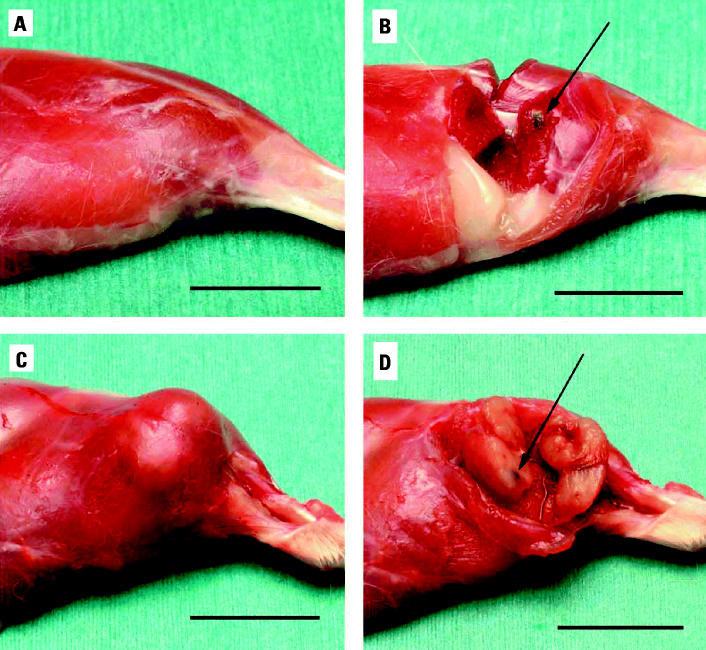
Effect of implanted WA pellets on F344 rats. (*A*) Gross appearance of Ta-implanted hind leg. (*B*) Dissected area around implanted Ta pellet (arrow indicates pellet). (*C*) Gross appearance of WA-implanted hind leg with tumor(s). (*D*) Dissected area around implanted WA pellet with tumor surrounding pellet (arrow indicates pellet). Bar = 2 cm.

Upon euthanasia, the animals underwent necropsy, and tissue samples were taken for various analyses. Figure 2 shows the appearance of the hind limb of rats implanted with Ta (Figure 2A) or WA (Figure 2C) for 26 and 23 weeks, respectively, before surgical removal of the implanted pellets. The gross anatomy of the Ta-implanted leg is normal, whereas in the WA leg the tumor is clearly visible. Upon dissection, no obvious abnormalities were observed in the Ta-implanted animals, and the pellets could be easily removed (Figure 2B). However, in the WA-implanted animals, the pellets were surrounded by tumor (Figure 2D). In many cases, the interior of the tumor had become necrotic and/or hemorrhagic. Similar tumors were found for both WA- and Ni-implanted animals. In low-dose WA animals, tumors were found surrounding the WA pellets only. No tumors were found surrounding implanted Ta pellets. Implanted WA pellets rapidly oxidized and had a slightly eroded appearance. Ta pellets did not have an eroded appearance even after implantation for 6 months. However, despite their appearance, the WA pellets lost < 5% of their mass over this time.

**Figure 3:**
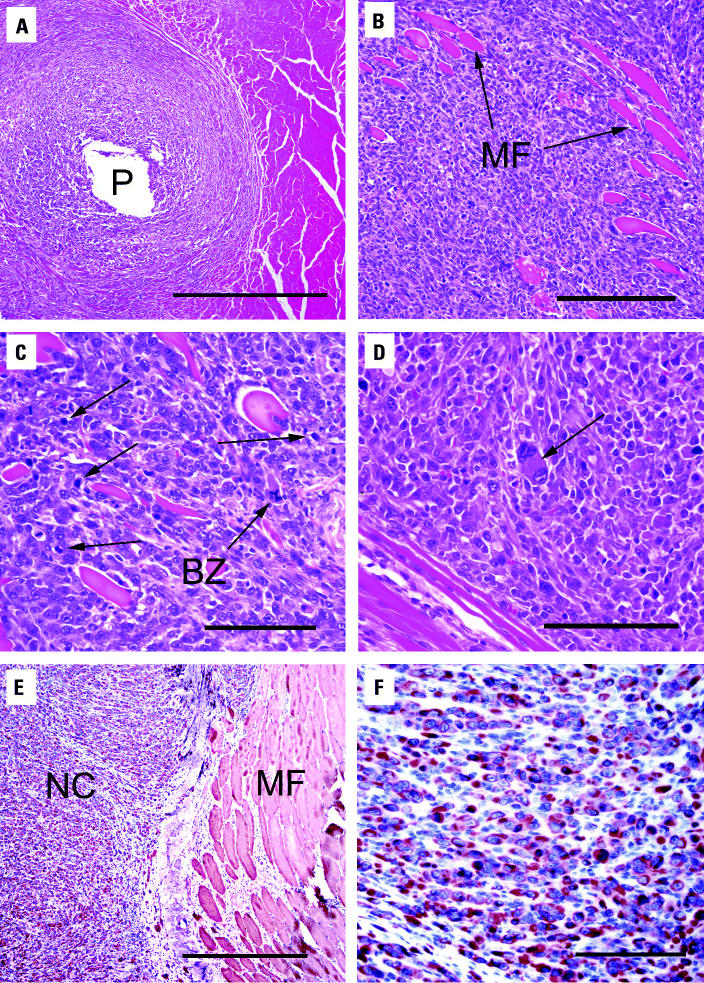
Histopathologic examination of leg tumor surrounding WA pellet. (*A*) H&E-stained section of leg tumor from F344 rat showing WA pellet hole (P); bar = 500 μm. (*B*) H&E-stained tumor section showing neoplastic infiltration of preexisting muscle fibers (MF); bar = 200 μm. (*C*) H&E-stained tumor section showing neoplastic cells with numerous mitoses (arrows) and bizarre mitotic figures (BZ); bar = 100 μm. (*D*) H&E-stained tumor section showing pleomorphic cell (arrow); bar = 100 μm. (*E*) Desmin staining of leg tumor showing neoplastic cells (NC) and muscle fibers (MF); bar = 500 μm. (*F*) Desmin staining of neoplastic cells; bar = 50 μm.

Tumor tissue was histopathologically examined and characterized. Figure 3A shows the neoplastic cells surrounding the site of the implanted WA pellet. These cells infiltrated preexisting skeletal muscle fibers. Fibers that became isolated by this process degenerated and demonstrated a loss of cross-striations and internalization of nuclei (Figure 3B,C). Neoplastic cells were pleomorphic with marked anisocytosis and anisokaryosis (Figure 3D). In addition, an extremely high mitotic rate was observed in these cells, and bizarre mitoses were present. Immunohistochemical staining was used to determine the origin of these neoplastic cells. The cells were strongly positive for desmin (Figure 3E,F), suggesting a skeletal muscle origin.

**Figure 4:**
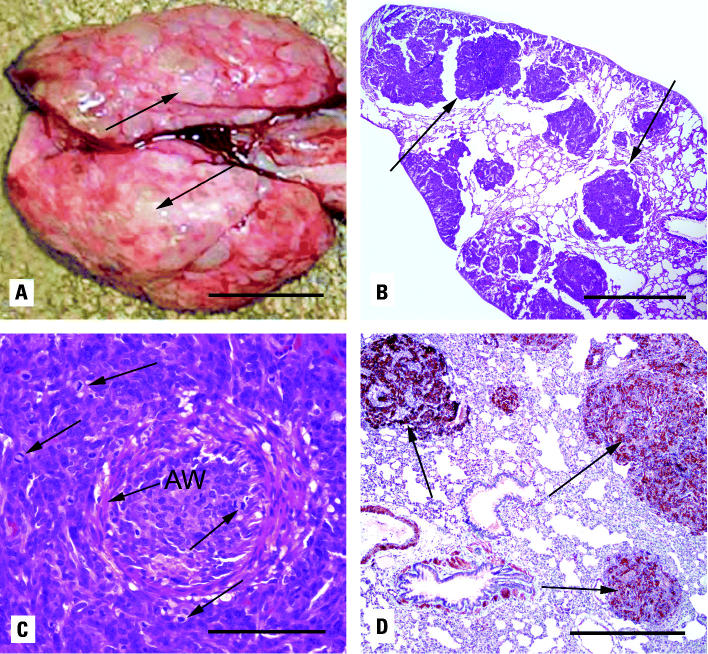
Lung metastases from WA-implanted F344 rats. (*A*) Gross appearance of pulmonary metastases from WA-implanted rat (arrows indicate metastatic foci); bar = 1 cm. (*B*) H&E-stained section of pulmonary metastases (arrows); bar = 1 mm. (*C*) H&E-stained section of an occluded pulmonary arteriole [arrow indicates vascular smooth muscle wall (AW)] showing neoplastic cells with numerous mitoses (arrows); bar = 50 μm. (*D*) Desmin staining of pulmonary metastases (arrows); bar = 500 μm.

In the WA-implanted animals, the tumors had metastasized to the lung. None of the Ni-implanted animals showed signs of lung metastases, although some exhibited endogenous histiocytic lipid pneumonia not seen in the WA animals. Figure 4A shows numerous metastatic foci in the lungs of a high-dose WA rat. These multiple masses obscure > 50% of the lung surface and up to 90% in the latter stages of development. Figure 4B shows a photomicrograph of these pulmonary metastases. Apparent is the multifocal, vascular orientation of these neoplasms. There are neoplastic cells surrounding the arterioles and bronchioles, expanding the alveolar septae, and replacing alveolar spaces. These neoplastic cells have a high mitotic rate and are often seen surrounding or occluding arterioles (Figure 4C). Figure 4D shows that the metastatic neoplastic cells, as well as vascular and airway smooth muscle, are strongly positive for the muscle marker desmin.

Selected hematologic and organ weight parameters for euthanized rats are shown in Table 1. The Ta data were obtained from rats implanted with Ta pellets for 6 months. The data for the remaining groups were obtained at the time the rats became moribund because of tumor development. No significant differences in organ/body weight ratios were seen for the low-dose WA- or Ni-implanted animals compared with Ta-implanted control rats. However, high-dose WA-implanted rats showed significantly higher spleen:body weight ratios compared with control rats. In addition, thymus:body weight ratios were decreased in the high-dose WA rats. Because the spleen and thymus are integral components of the immune system, these changes suggest that embedded WA, at certain levels, may be immunotoxic. The kidney:body weight ratio for high-dose WA rats was also significantly higher than that of Ta-implanted rats. High-dose WA rats euthanized 1 and 3 months after pellet implantation also exhibited significantly elevated spleen:body weight ratios compared with the appropriate Ta-implanted control rats (Tables 2 and 3). Thymus:body weight ratios, however, were not significantly different. At 3 months post-implantation, the kidney:body weight ratio in high-dose WA rats was significantly higher than that in Ta rats, but it was significantly lower at 1 month postimplantation. There were no 1- and 3-month Ni-implanted groups.

WA-implanted animals had significant changes in a number of hematologic parameters. Rats implanted with 20 WA pellets exhibited significant increases in white blood cell counts, red blood cell counts, hemoglobin, and hematocrit levels compared with Ta control rats, whereas rats implanted with 20 Ni pellets had significant decreases in red blood cell counts, hemoglobin, and hematocrit levels (Table 1). Hematologic parameters from low-dose WA rats were not statistically different from controls. Statistically significant increases in red blood counts, hemoglobin, and hematocrit levels were observed in high-dose WA animals as early as 1 month after pellet implantation and persisted throughout the experimental period (Tables 2 and 3). In addition, there were statistically significant increases in the numbers of neutrophils, lymphocytes, monocytes, and eosinophils present in high-dose WA animals. Low-dose WA animals had elevated neutrophil, lymphocyte, and monocyte numbers at 3 months post-implantation, but only the neutrophil numbers were statistically different from the controls at the 5–6 month euthanasia point. The Ni-implanted animals had significantly lower lymphocyte counts than the controls. All other parameters were statistically identical to the controls. These results suggest there is a dose-dependent perturbation in many hematology parameters as a result of an increasing WA pellet number.

## Discussion

Tungsten-based alloys are currently being used as replacements for DU in kinetic-energy penetrators and for lead in small-caliber ammunition. However, the health effects of these unique alloys have not been investigated, especially in the case of embedded fragments such as shrapnel wounds. In this study, using male F344 rats and a system designed to investigate the effects of embedded metal fragments (AFRRI 1996), we have shown the embedded weapons-grade WA (91.1% W, 6.0% Ni, 2.9% Co) results in rapid tumor formation at the implantation site in 100% of the rats. The rate of tumor formation correlates with pellet number. Ni-implanted rats also develop tumors at the implantation site, although not as rapidly as seen with WA. Histopathologic and immunohistochemical data support a diagnosis of a pleomorphic rhabdomyosarcoma for both the WA- and Ni-induced leg tumors (Altmannsberger et al. 1985).

Rats implanted with 20 WA pellets (high-dose WA) showed significantly increased spleen:body weight ratios compared with Ta control rats. Low-dose WA rats (four WA pellets) also exhibited increased spleen:body weight ratios, but these increases were not statistically significant (ANOVA followed by Dunnett’s test). Values for Ni-implanted rats were identical to control rats. The spleen changes observed in the high-dose WA rats were apparent as early as 1 month after pellet implantation. Once again, low-dose WA rats showed increased, but not statistically significant, spleen:body weight ratios. With the exception of the spleen, the only other organ:body weight perturbations were seen in high-dose WA rats and included a decrease in thymus:body weight ratio at approximately 5 months and changes in kidney:body weight ratios. The 1-month kidney:body weight ratio for high-dose WA rats was significantly lower than control. However, from 3 months on, these ratios were significantly higher than control. It is possible that the lower kidney weights at 1 month postimplantation represent a toxic response to the heavy metals from the implanted pellets, but by 3 months and later, the kidney has begun to respond in a different manner. Although there were no gross abnormalities of the kidney at necropsy, we continue to investigate this observation.

A variety of hematologic changes were observed in WA- and Ni-implanted rats. Ni-implanted rats showed a significant decrease in red blood cells, hemoglobin, and hematocrit at the time of morbidity, indicating possible Ni-induced anemia. For low-dose WA rats the hematologic changes, including significant increases in red blood cells, white blood cells, hemoglobin, hematocrit, neutrophils, lymphocytes, and monocytes, peaked at 3 months postimplantation and returned to normal by 5–6 months. High-dose WA rats demonstrated the same changes observed in low-dose WA rats, but they occurred much more rapidly (as early as 1 month postimplantation) and persisted throughout the life of the animal. The splenomegaly and hematologic changes observed in these rats are suggestive of polycythemia. Cobalt has been used experimentally to induce polycythemia in rats (Endoh et al. 2000; Rakusan et al. 2001), although the concentration required is far greater than found in the WA pellets. In addition, the speed at which these hematologic changes occurred in the high-dose WA rats was also surprising. These results suggest a dose-dependent perturbation in many hematology parameters as a result of an increasing WA pellet number.

The search for munitions that are considered environmentally friendly yet still retain their military effectiveness has led to the appearance of many unique alloys on the modern battlefield. Often, decisions on the health consequences of exposure (inhalation, ingestion, wound contamination, etc.) to these specific alloys are based on studies that investigated only one specific metal of the alloy rather than the particular alloy in question. Tungsten-based munitions are a recent addition to many countries’ arsenals, primarily in response to the continuing concerns regarding the potential environmental and health effects of DU in kinetic-energy penetrators and of lead in small-caliber ammunition. For years, exposure to tungsten was thought to be of little consequence to health. In fact, tungsten is occasionally found as a minor component in some of the various alloys used to produce medical implant devices such as artificial hips and knees. The tungsten concentration in these alloys ranges from 5% to 15%. Because the alloy used in WA munitions usually contains > 90% tungsten, along with smaller amounts of other metals, it was also assumed that exposure to these alloys would present little or no health risk. As we have shown here, this is not the case in our rodent model. Embedded WA pellets not only resulted in aggressive, metastatic, pleomorphic rhabdomyosarcomas, but also caused significant hematopoietic changes well before the carcinogenic effect was observed. It seems unlikely that these adverse health effects can be attributed solely to the small amounts of Ni and/or Co present in the alloy. The tumors induced by the 100% Ni implants occurred later than those induced by the alloys containing 6% Ni. However, recent *in vitro* studies have demonstrated a synergistic effect in terms of damage when tungsten is present with these metals (Miller et al. 2001, 2002).

The mechanism of the effects reported here with embedded WA pellets remains unclear. Despite the fact that the smooth and impermeable surface of the pellets represent characteristics known capable of inducing foreign-body or solid-state carcinogenesis (Bates and Klein 1966; Brand et al. 1975), this process is unlikely to have occurred in our experiments because implanted Ta pellets of an identical geometry and surface resulted in no tumor formation. One possibility is that free-radical reactions at the interface of the pellet and tissue could result in damage leading to carcinogenesis. Recently, the role of tungsten in human health and disease has come under increased scrutiny. Environmental testing of the leukemia cluster around Fallon, Nevada, in the United States showed slightly elevated levels of several heavy metals including uranium and Co but significantly elevated levels of tungsten [Centers for Disease Control and Prevention (CDC) 2003]. Although no definitive link between elevated tungsten levels and cancer has been established, because of the uncertainty surrounding this issue, the U.S. National Toxicology Program recently added tungsten to their list of compounds to be assessed for adverse health effects. Further study of the health effect of tungsten and WAs is clearly indicated.

## Figures and Tables

**Table 1:** Selected hematologic and organ weight parameters (mean ± SEM) for euthanized rats.

	Ta	WA (low)	WA (high)	Ni
White blood cells (10^3^/μL)	3.19 ± 0.24	3.95 ± 0.43	4.56 ± 0.29*	2.56 ± 0.20
Red blood cells (10^6^/μL)	8.32 ± 0.09	8.03 ± 0.19	10.10 ± 0.07**	7.46 ± 0.13**
Hemoglobin (g/dL)	14.50 ± 0.13	13.90 ± 0.36	16.46 ± 0.30**	12.95 ± 0.23**
Hematocrit (%)	41.77 ± 0.53	40.38 ± 0.96	50.18 ± 0.39**	38.12 ± 0.77**
MCV (fL)	50.22 ± 0.16	50.26 ± 0.28	49.71 ± 0.16	51.08 ± 0.66
MCH (pg)	17.46 ± 0.15	17.31 ± 0.13	16.30 ± 0.28**	17.35 ± 0.08
MCHC (g/dL)	34.77 ± 0.36	34.46 ± 0.32	32.81 ± 0.62**	34.05 ± 0.50
RDW (%)	12.54 ± 0.09	13.07 ± 0.11**	13.77 ± 0.09**	13.04 ± 0.16*
Platelets (10^3^/μL)	562.00 ± 14.72	542.05 ± 14.27	467.50 ± 17.57**	487.18 ± 26.10*
MPV (fL)	9.93 ± 0.69	8.64 ± 0.52	10.13 ± 0.62	8.97 ± 0.52
Neutrophils (10^3^/μL)	0.79 ± 0.05	1.03 ± 0.09*	1.31 ± 0.12**	0.78 ± 0.09
Lymphocytes (10^3^/μL)	2.21 ± 0.18	2.42 ± 0.17	2.95 ± 0.23*	1.63 ± 0.12*
Monocytes (10^3^/μL)	0.07 ± 0.01	0.09 ± 0.02	0.13 ± 0.02*	0.05 ± 0.01
Eosinophils (10^3^/μL)	0.08 ± 0.01	0.08 ± 0.01	0.12 ± 0.01**	0.06 ± 0.01
Basophils (10^3^/μL)	0.02 ± 0.00	0.03 ± 0.00	0.03 ± 0.00	0.02 ± 0.00
Spleen (mg/g bw)	2.18 ± 0.10	2.30 ± 0.08	2.60 ± 0.06**	2.17 ± 0.05
Thymus (mg/g bw)	0.86 ± 0.03	0.76 ± 0.04	0.70 ± 0.04*	0.74 ± 0.07
Liver (mg/g bw)	29.21 ± 0.28	29.39 ± 0.24	28.77 ± 0.35	29.52 ± 0.39
Kidney (mg/g bw)	5.13 ± 0.06	5.13 ± 0.06	5.36 ± 0.05*	5.15 ± 0.08
Testes (mg/g bw)	7.31 ± 0.07	7.20 ± 0.08	7.40 ± 0.10	7.21 ± 0.14

Abbreviations: bw, body weight; MCH, mean corpuscular hemoglobin; MCHC, mean corpuscular hemoglobin concentration; MCV, mean corpuscular volume; MPV, mean platelet volume; RDW, red blood cell distribution width. Data represent mean ± SEM of 20 observations (10 for Ni group).

* *p* < 0.05, and

** *p* < 0.01 compared with the Ta control group by one-way ANOVA followed by Dunnett’s test for group mean comparisons.

**Table 2:** Selected hematologic and organ weight parameters (mean ± SEM) for rats implanted with metal pellets for 3 months.

	Ta	WA (low)	WA (high)
White blood cells (10^3^/μL)	2.88 ± 0.20	4.06 ± 0.14**	4.01 ± 0.21**
Red blood cells (10^6^/μL)	7.48 ± 0.06	8.48 ± 0.15*	9.10 ± 0.70**
Hemoglobin (g/dL)	12.90 ± 0.09	15.48 ± 0.35*	17.29 ± 0.15**
Hematocrit (%)	38.10 ± 0.27	42.14 ± 0.73*	44.79 ± 0.62**
MCV (fL)	50.96 ± 0.45	49.70 ± 0.09	48.87 ± 0.39
MCH (pg)	17.26 ± 0.12	18.27 ± 0.17	17.65 ± 0.12
MCHC (g/dL)	33.84 ± 0.35	36.71 ± 0.31**	35.89 ± 0.31**
RDW (%)	12.82 ± 0.33	12.68 ± 0.12	13.61 ± 0.09**
Platelets (10^3^/μL)	513.20 ± 38.36	585.11 ± 35.87	568.29 ± 8.82
MPV (fL)	9.58 ± 1.13	9.14 ± 0.59	11.74 ± 0.51
Neutrophils (10^3^/μL)	0.62 ± 0.04	0.79 ± 0.03*	0.91 ± 0.08*
Lymphocytes (10^3^/μL)	2.10 ± 0.16	3.06 ± 0.14*	2.82 ± 0.17*
Monocytes (10^3^/μL)	0.04 ± 0.01	0.07 ± 0.01*	0.08 ± 0.01*
Eosinophils (10^3^/μL)	0.09 ± 0.01	0.09 ± 0.01	0.09 ± 0.01
Basophils (10^3^/μL)	0.01 ± 0.00	0.01 ± 0.00	0.02 ± 0.00
Spleen (mg/g bw)	2.07 ± 0.03	2.16 ± 0.03	2.50 ± 0.03**
Thymus (mg/g bw)	0.73 ± 0.03	0.84 ± 0.03	0.70 ± 0.04
Liver (mg/g bw)	30.58 ± 0.33	31.00 ± 0.33	30.27 ± 0.31
Kidney (mg/g bw)	5.43 ± 0.06	5.73 ± 0.23	5.76 ± 0.04**
Testes (mg/g bw)	8.34 ± 0.12	8.21 ± 0.46	8.42 ± 0.18

Abbreviations: bw, body weight; MCH, mean corpuscular hemoglobin; MCHC, mean corpuscular hemoglobin concentration; MCV, mean corpuscular volume; MPV, mean platelet volume; RDW, red blood cell distribution width. Data represent mean ± SEM of 15 observations.

* *p* < 0.05, and

** *p* < 0.01 compared with the age-matched Ta control group by one-way ANOVA followed by Dunnett’s test for group mean comparisons.

**Table 3:** Selected hematologic and organ weight parameters (mean ± SEM) for rats implanted with metal pellets for 1 month.

	Ta	WA (low)	WA (high)
White blood cells (10^3^/μL)	3.86 ± 0.20	3.81 ± 0.14	3.86 ± 0.21
Red blood cells (10^6^/μL)	7.84 ± 0.08	7.74 ± 0.07	8.50 ± 0.07**
Hemoglobin (g/dL)	13.65 ± 0.15	14.81 ± 0.16	15.84 ± 0.14**
Hematocrit (%)	40.15 ± 0.42	39.66 ± 0.50	43.29 ± 0.35**
MCV (fL)	51.20 ± 0.14	51.22 ± 0.31	50.98 ± 0.19
MCH (pg)	17.41 ± 0.05	19.12 ± 0.09	18.64 ± 0.19**
MCHC (g/dL)	34.01 ± 0.12	37.37 ± 0.29	36.56 ± 0.41**
RDW (%)	12.21 ± 0.11	12.69 ± 0.11	14.18 ± 0.18**
Platelets (10^3^/μL)	646.50 ± 18.76	641.00 ± 17.97	756.20 ± 43.48*
MPV (fL)	7.91 ± 0.40	8.56 ± 0.39	9.90 ± 0.55*
Neutrophils (10^3^/μL)	0.65 ± 0.04	0.79 ± 0.05	0.81 ± 0.04**
Lymphocytes (10^3^/μL)	3.04 ± 0.18	2.85 ± 0.13	2.90 ± 0.18
Monocytes (10^3^/μL)	0.06 ± 0.00	0.06 ± 0.01	0.07 ± 0.00
Eosinophils (10^3^/μL)	0.07 ± 0.01	0.08 ± 0.01	0.05 ± 0.00*
Basophils (10^3^/μL)	0.02 ± 0.00	0.02 ± 0.00	0.01 ± 0.00
Spleen (mg/g bw)	2.37 ± 0.06	2.42 ± 0.05	2.73 ± 0.04**
Thymus (mg/g bw)	1.07 ± 0.03	1.14 ± 0.04	1.06 ± 0.03
Liver ((mg/g bw)	34.47 ± 0.26	34.31 ± 0.22	34.18 ± 0.61
Kidney (mg/g bw)	6.17 ± 0.08	6.06 ± 0.06	5.91 ± 0.05*
Testes (mg/g bw)	10.10 ± 0.16	9.86 ± 0.13	9.98 ± 0.11

Abbreviations: bw, body weight; MCH, mean corpuscular hemoglobin; MCHC, mean corpuscular hemoglobin concentration; MCV, mean corpuscular volume; MPV, mean platelet volume; RDW, red blood cell distribution width. Data represent mean ± SEM of 15 observations.

* *p* < 0.05, and

** *p* < 0.01 compared with the age-matched Ta control group by one-way ANOVA followed by Dunnett’s test for group mean comparisons.
